# The Biomechanical Effect of the Sagittal Split Ramus Osteotomy on the Temporomandibular Joint: Current Perspectives on the Remodeling Spectrum

**DOI:** 10.3389/fphys.2019.01021

**Published:** 2019-08-07

**Authors:** Pieter-Jan Verhelst, Fréderic Van der Cruyssen, Antoon De Laat, Reinhilde Jacobs, Constantinus Politis

**Affiliations:** ^1^OMFS IMPATH Research Group, Department of Imaging and Pathology, Faculty of Medicine, KU Leuven, Leuven, Belgium; ^2^Department of Oral and Maxillofacial Surgery, University Hospitals Leuven, Leuven, Belgium; ^3^Department of Oral Health Sciences, KU Leuven, Leuven, Belgium; ^4^Department of Dentistry, University Hospitals Leuven, Leuven, Belgium; ^5^Department of Dental Medicine, Karolinska Institutet, Stockholm, Sweden

**Keywords:** orthognathic surgery, sagittal split ramus osteotomy, temporomandibular joint, degenerative joint disease, condylar resorption

## Abstract

The sagittal split ramus osteotomy is a key approach for treating dentofacial deformities. Although it delivers excellent results, the sagittal split ramus osteotomy is believed to induce stress to the temporomandibular joint. Potential stress inducers could be classified as intra- and postoperative factors resulting in an inflammatory response and molecular cascades, which initiate physiological remodeling. Occasionally, this process exceeds its capacity and causes pathological remodeling, through either degenerative joint disease or condylar resorption. Hard evidence on how orthognathic surgery causes inflammation and how this inflammation is linked to the spectrum of remodeling remains scarce. Current concepts on this matter are mainly based on clinical observations and molecular mechanisms are extrapolated from fundamental research in other body parts or joints. This perspective study provides an overview of current knowledge on molecular pathways and biomechanical effects in temporomandibular joint remodeling. It provides research directions that could lead to acquiring fundamental evidence of the relation of orthognathic surgery and inflammation and its role in remodeling. Performing osteotomies in animal models and identifying inflammatory mediators as well as their effect on the joint seem promising. Patients affected by pathological remodeling can also provide samples for histological as well as molecular analysis. Individual susceptibility analysis by linking certain suspect phenotypes to genetic variation could identify the cause and molecular pathway responsible for degenerative joint disease and condylar resorption, ultimately leading to clinically applicable treatment and prevention strategies.

## Introduction

Orthognathic surgery plays a crucial role in the treatment of dentofacial deformities. After performing an osteotomy in the desired jaw, the jaw is mobilized and fixed into its planned position. The sagittal split ramus osteotomy (SSRO) in the mandible was introduced in 1957 by Obwegeser (Trauner and Obwegeser, 1957), and many modifications were later described (Böckmann et al., 2014). The basic features, however, remained unchanged: the proximal condylar-bearing bone plate is separated from the tooth-bearing segment. This necessitates a broad healing area at the separation site, which is under direct view of the surgeon. An SSRO also induces changes at the temporomandibular joint (TMJ), which is not under direct view of the surgeon. Clincial methods available for judging the condition of the TMJ are postoperative anamnestic data and clinical symptoms, complemented with radiographic signs detected with panoramic radiography (PAN), cone beam computed tomography (CBCT), and magnetic resonance imaging (MRI). In some patients, arthroscopy allows a direct view of the condition of the upper joint compartment.

It is believed that the SSRO causes biomechanical stress at the TMJ. This biomechanical stress leads to a process frequently observed on follow-up imaging called physiological joint remodeling (Hoppenreijs et al., 1998; de Assis Ribeiro Carvalho et al., 2010; Catherine et al., 2016; Gomes et al., 2017; Xi et al., 2017; Vandeput et al., 2019). When joint remodeling surpasses its physiological capacity, pathological remodeling can occur, regardless of whether the patient had a pre-existing TMJ dysfunction. Two potential outcomes are feared (Jung et al., 2015; Politis et al., 2018), as follows:

1. Degenerative joint disease (DJD), which leads to a gradual destruction of the articular disc and articular surfaces. DJD causes symptoms of pain, limited mouth opening, and joint sounds.
2. Condylar resorption, which is characterized by the rapid loss of ramus and condylar height and overall condylar volume. This condition leads to mandibular retrognathia, which manifests as a loss of posterior facial height and a frontal open bite. Joint function is often preserved, and symptoms, like pain and joint sounds, might be absent.

Both clinical entities have highly variable prevalences reported in literature. Postoperative onset of temporomandibular disorders that can progress to DJD is reported between 6.7 and 25% (Panula and Somppi, 2000; Dervis and Tuncer, 2002). Postoperative condylar resorption rates range from 1 to 31% (Catherine et al., 2016; Mousoulea et al., 2016). These pathological remodeling processes have a similar origin, but they evolve into distinct entities through molecular pathways yet unknown.

This study provides hypotheses on how orthognathic surgery causes biomechanical stress at the TMJ and how this stress can initiate inflammation in the TMJ. It provides possible links between inflammation and the remodeling spectrum of the joint. In the end, research directions are suggested to test these hypotheses.

## The Biomechanical Effect of Orthognathic Surgery on the Temporomandibular Joint

**Figure 1:**
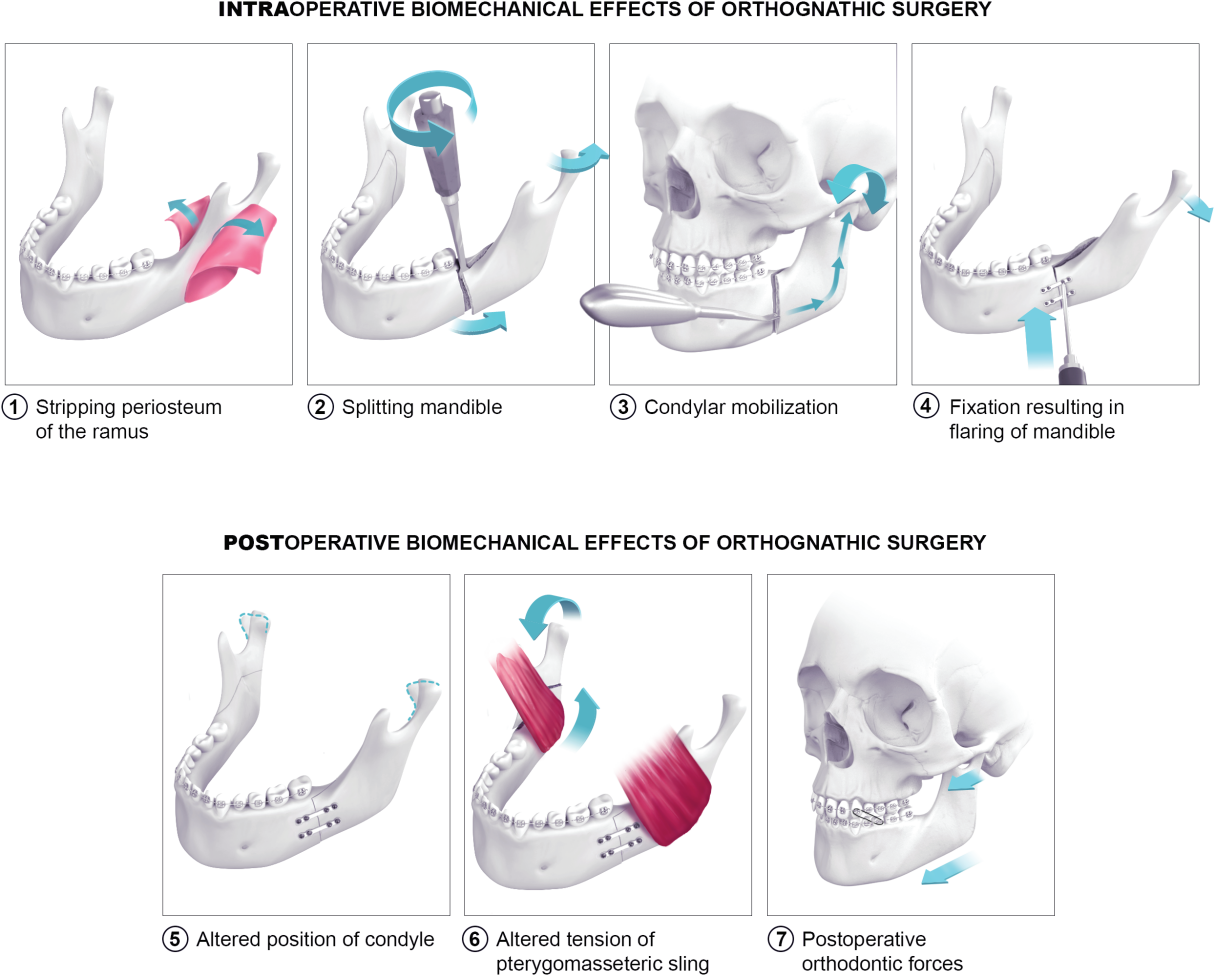
Biomechanical effects of orthognathic surgery on the temporomandibular joint. Scenes 1–4 depict probable intraoperative biomechanical effects of orthognathic surgery on the temporomandibular joint. Scenes 5–7 illustrate postoperative biomechanical effects.

Orthognathic surgery, especially the SSRO, is believed to induce biomechanical stress at TMJ (Figure 1). During surgery, four procedures can cause potential biomechanical stress: stripping the periosteum, performing the mandibular split, mobilizing the condyle in the fossa, and fixing the new position of the tooth-bearing segment. During surgery, stripping the periosteum of the ramus causes temporary devascularization and denervation of the ramus. Orthopedic experimental literature has shown that this procedure increases the risk of cortical bone loss (Mercurio et al., 2012). It is possible that periosteal stripping might also cause bone loss at the level of the TMJ. During the mandibular split, rotational forces are applied at the split site. These forces can exert stress, because cantilever force at the joint can put pressure on the condyle in its fossa. Next, before fixing the distal segment in the desired position, the condyle-bearing segment must be seated in the fossa to avoid condylar sag. Therefore, the proximal part of the osteotomized mandible is pushed into the fossa. This condylar mobilization might induce an injury at the articular surfaces (Jung et al., 2015). Finally, the distal segment is fixed to the proximal segment after determining its desired position. Currently, fixation can be performed with mini-plates and monocortical screws, with bicortical screws without mini-plates, or with a combination of these techniques. These methods are meant to be performed in a passive setting after correctly positioning the proximal and distal segments (Joss and Vassalli, 2008). However, eccentric placement of the screws can render the fixation “active” and induce condylar torque (Arnett and Gunson, 2013).

In the postoperative phase, three types of biomechanical stress are possible. The first is a prolonged alteration of the condyle position in the fossa, induced by peri-operative maneuvers (Gomes et al., 2017). The altered position of the condyle can create pressure at the articular surface. Second, displacement of osteotomized segments may increase tension in the muscles attached to the mandible. The stretched muscles, such as the pterygomasseteric sling, will then exert force on the osteotomized segments, which can be transferred to the TMJ (Byeon et al., 2013). Third, it is believed that heavy postoperative orthodontic forces incurred with the use of elastics can also induce mechanical stress at the TMJ (Handelman and Greene, 2013).

All these events are believed to play a role in increasing the biomechanical load on the TMJ. These are all probable factors of which it is yet unknown how big their individual role is in inducing mechanical overloading. As studies in other joints have shown that mechanical overloading leads to frank inflammation (Sturmer, 2004; Berenbaum, 2013), it is hypothesized that orthognathic surgery causes a similar situation in the TMJ. Clinically, an inflammatory response in the joint is sometimes detected in the initial postoperative period as a transient postoperative class III occlusion, caused by joint edema. The prevailing hypothesis is that an overload-induced inflammation will initiate physiological postoperative remodeling. Each condyle and fossa reacts adaptively during the postoperative remodeling phase, until a new biomechanical equilibrium is achieved (de Assis Ribeiro Carvalho et al., 2010; Franco et al., 2013; Gomes et al., 2017; Xi et al., 2017). Although this hypothesis seems likely, literature lacks evidence on the biochemical identification and quantification of inflammation of the TMJ in orthognathic patients.

**Figure 2:**
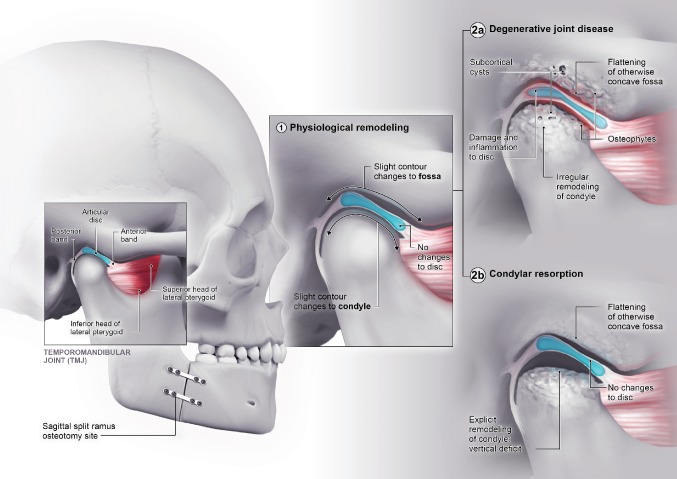
Spectrum of temporomandibular joint remodeling following orthognathic surgery. The normal anatomy of the joint is presented. Following orthognathic surgery, physiological remodeling (1) of the joint occurs. In some cases, this will evolve in pathological remodeling (2). The anatomical characteristics of degenerative joint disease (2a) and condylar resorption (2b) are exemplified.

In some cases, the physiological remodeling capacity reaches a limit and transgresses to pathological, regressive remodeling (Figure 2; Gunson et al., 2012; Murphy et al., 2013). This can either result in DJD or condylar resorption, two known clinical entities and feared outcomes of orthognathic surgery. DJD is characterized by pathological irregular bone remodeling and damage to the soft tissues of the joint. Two major questions remain regarding these entities. First, it is unclear when and why physiological remodeling reaches its limits and becomes pathological. Second, although DJD and condylar resorption have a common origin (i.e., a biomechanical overload to the joint), their outcomes are quite different. The question of why one overloaded joint develops DJD and another develops condylar resorption remains an enigma. The actual molecular pathways of these entities and their link with inflammation caused by orthognathic surgery should provide further insight into these questions.

## Degenerative Joint Disease of the Temporomandibular Joint

The physiology of DJD is well studied in the TMJ and other joints. Repetitive mechanical overloading of the joint leads to increased shear stress at the articular surfaces and elevated intra-articular pressure. When the intra-articular pressure exceeds the capillary perfusion pressure, a local zone of hypoxia is created. When the pressure normalizes, a hypoxia-reperfusion injury occurs with the production of free radicals (Kawai et al., 2008). These free radicals have multiple effects. First, they promote the degradation of peripheral lubricants, such as hyaluronic acid, which results in further increasing the mechanical stress on the articular surface (Kawai et al., 2008). They also promote the formation of adhesions on articular surfaces through molecular crosslinking (Dijkgraaf et al., 2003). Finally, they induce the production of inflammatory cytokines by stimulating gene transcription (Fukuoka et al., 1993). Cytokines are believed to play an important role in the inflammatory pathway leading to DJD (Puzas et al., 2001; Tanaka et al., 2008; Vernal et al., 2008). Where the upregulation of proinflammatory cytokines has been identified in human studies, most evidence on the role of free radicals in DJD is derived from *in vitro* or animal studies.

Chondrocytes also play an important role in the pathogenesis of DJD (van der Kraan and van den Berg, 2012). In the articular cartilage, they are embedded in a highly hydrated extracellular matrix (ECM); indeed, the wet weight of the ECM is 75% water. The dry weight of this ECM consists mostly of collagen and proteoglycans, such as hyaluronic acid. Chondrocytes respond to a mechanical overload in the joint by stimulating reactions in the articular cartilage and in the subchondral bone (van der Kraan and van den Berg, 2012). In the cartilage, mechanical overloading induces chondrocyte differentiation, which leads to hypertrophy. Animal experiments have identified WNT5A as a crucial signal molecule of the cartilage-subchondral bone unit and with a major role in upregulation of the chondrocytes (Ge et al., 2017). These hypertrophic chondrocytes start secreting DJD-promoting molecules, such as matrix metalloproteases (MMPs) (Fukui et al., 2008), vascular endothelial growth factor (VEGF) (Forsythe et al., 1996; Tanaka et al., 2005), inflammatory cytokines, and chemokines (Puzas et al., 2001; Tanaka et al., 2008). MMPs are proteases that use zinc as a cofactor; they degrade the ECM of cartilage. MMPs 1, 2, 3, 9, and 13 are known to play a role in TMJ DJD (Pufe et al., 2004b; Kurz et al., 2005). They primarily degrade collagen and proteoglycans. This process is regulated by natural tissue inhibitors of MMPs. In affected TMJs, free radicals can oxidize these inhibitors, which hampers their inhibitory function. Thus, the regulation of MMP activity is disturbed, and cartilage degradation is favored (Wong et al., 2003).

Currently, it is recognized that DJD is not solely a “cartilage disease,” but an entity that encompasses the whole joint, including the underlying bone. The subchondral bone plays an important role in DJD, and this role has long been underestimated. In response to mechanical loads, deep hypertrophic chondrocytes also produce VEGF, which diffuses downward, and predominantly affects the subchondral bone (Forsythe et al., 1996; Tanaka et al., 2005, 2008). There, in addition to endothelial recruitment, VEGF promotes osteoclast recruitment, osteoclast differentiation, and initiation of resorption. Consequently, VEGF increases the rate of bone turnover and subchondral plate resorption, which eventually leads to subchondral sclerosis (Mackie et al., 2008). VEGF also upregulates the function of MMPs, which promotes further degradation of the ECM (Pufe et al., 2004a). The reciprocal activities of osteoclasts and osteoblasts create bony bridges that reach into the cartilage. Newly formed blood vessels accompany these bridges, creating a vascular supply in the otherwise avascular cartilage (Mapp et al., 2008; Jiao et al., 2010). This gateway between subchondral bone and cartilage further facilitates the diffusion of inflammatory cytokines.

The pathological remodeling of the TMJ is based on a complex system of molecular crosstalk, and inflammatory cytokines play a crucial role (Puzas et al., 2001; Tanaka et al., 2008). These cytokines are produced by a wide range of cells, including chondrocytes and synovial fibroblasts, and they bind to specific cell receptors, which further propagates the inflammatory response and leads to the aforementioned processes. Some of the most important cytokines are interleukin-1 (IL1), IL6, IL10, IL17, tumor necrosis factor-alpha, and the receptor activator of nuclear factor kappa-Β ligand (Puzas et al., 2001; Tanaka et al., 2008; Vernal et al., 2008; Gunson et al., 2012). These cytokines upregulate the synthesis of MMPs, which leads to further breakdown of the ECM. These cytokines also have an effect on neurosensory projections to the TMJ. Cytokines stimulate the synthesis and release of proinflammatory neuropeptides, which cause further tissue degradation and inflammatory pain. Moreover, inflammatory cytokines directly stimulate neurosensory endings.

The pathways and molecules listed above induce the degeneration of the cartilage-bone unit with a focus on tissue degradation. As DJD progresses, bone apposition at the condylar margins can occur. Endochondral bone formation leads to the emergence of osteophytes. Transforming growth factor beta (TGF-Β) and bone morphogenic protein 2 (BMP2) have been identified to play a role in this process. The role of these osteophytes remains debatable with some claiming that they serve a stabilizing role in the remodeled TMJ (van der Kraan and van den Berg, 2007).

All these molecular pathways lead to a state known as degenerative TMJ disease. This disease is characterized by articular disc damage, articular surface damage, subchondral sclerosis, synovitis, and regressive remodeling of the joint, in general. The clinical phenotype is characterized by pain, limited TMJ mobility, and the emission of sounds during joint function. MRIs might show disc pathology, joint effusion, bone edema, or a dislocated disc (Tanaka et al., 2008; Pantoja et al., 2018). CBCT, CT, and PAN reveal a narrowing of the joint space, flattening of the condyle and fossa, and a change in the inclination of the condyle. Arthroscopic evaluations might show signs of synovitis and chondromalacia (Stegenga et al., 1993).

## Condylar Resorption

Although some pathways that lead to DJD have been identified, the exact molecular pathway of condylar resorption remains fairly unknown. Condylar resorption is a rare postoperative complication that occurs in a small subset of patients during orthognathic surgery, but it causes profound consequences (Catherine et al., 2016; Mousoulea et al., 2016). Similar to DJD, condylar resorption is a pathological outcome of postoperative maladaptive TMJ remodeling (Gunson et al., 2012; Arnett and Gunson, 2013). However, the type of remodeling and the clinical phenotype differ from DJD, which suggests that the pathophysiology should also differ. Postoperative radiograph studies have shown that condylar resorption is characterized by a rapid loss of vertical height in the mandibular condyle and ramus. The end result is an incremental loss in vertical condylar and ramus height and an overall decrease in condylar volume (Hatcher, 2013; Hoppenreijs et al., 2013). This loss of volume can occur on all sides of the condyle, in contrast to the mostly superior flattening observed in DJD. Also, subcortical cysts and osteophyte formations are absent in condylar resorption (Hatcher, 2013). These features translate into pathognomonic symptoms of mandibular retrognathia, a loss of posterior facial height, and the emergence of a frontal open bite. In contrast to DJD, TMJ pain, and functional deficits are less prominent, which supports the hypothesis that the synovitis aspect of DJD is less obvious or even absent in condylar resorption (Mitsimponas et al., 2018). This hypothesis is further supported by the absence of reports that describe joint effusion in condylar resorption cases.

Where molecular pathways are being unraveled for DJD, our current knowledge regarding condylar resorption is minimal. As the initial stimulus of condylar resorption is the same as in DJD, a biomechanical overload on the joint, we could speculate that some of the same mechanisms are initiated in DJD and condylar resorption. Although there are some key differences to be noted. When evaluating radiographs of condylar resorption patients, joint spaces between articular surfaces of the joint are often preserved, suggesting that the articular cartilage is not degraded during the process. Mitsimponas et al. (Mitsimponas et al., 2018) hypothesized that the pathophysiology in condylar resorption is indeed rather focused in the subchondral bone and resorption acts as an aggressive adaptive measure to counteract the mechanical overload until sufficient vertical height is lost and the joint is “decompressed.” In condylar resorption joints, the main pathophysiological actor seems increased osteoclast activity and the cartilage, synovium, and articular disc seem less affected.

Some studies have suggested that the key explanation for the progression to condylar resorption instead of DJD is that different patients have different individual systemic susceptibilities. This individual susceptibility is characterized by certain clinical phenotypes that seem to be frequently associated with condylar resorption. For example, resorption has been mostly identified in young females; thus, it has been suggested that condylar resorption might be related to low estrogen levels, more specifically, 17 estradiol β (Gunson et al., 2012; Arnett and Gunson, 2013; Nicolielo et al., 2017). Estrogen receptors α and β have been found in the articular tissues of the TMJ, and different polymorphisms of these receptors can lead to different susceptibilities. Another hypothesis is that actual condylar architecture of these patients differs from healthy patients. The pathological focus should then lie in the signaling cascade that induces bone and cartilage formation. Bone morphogenetic proteins (BMPs) become molecules of interest in this case. These phenotypes are probably linked to certain genetic variations that are the cause of the individual susceptibility. Until now, this is still a hypothesis that needs to be tested in the case of pathological condylar remodeling. Other bone diseases such as osteoporosis are already being genetically defined (Karasik et al., 2016). Regarding the TMJ, a recent study identified genetic variations associated with temporomandibular disorders. This lead to the identification of a specific molecular pathway that plays a role its pathophysiology (Smith et al., 2019). The emerging accessibility of genetic testing with techniques such as comparative genome hybridization arrays and whole- or targeted-exome sequencing will provide further insight into this matter.

## Future Directions

**Figure 3:**
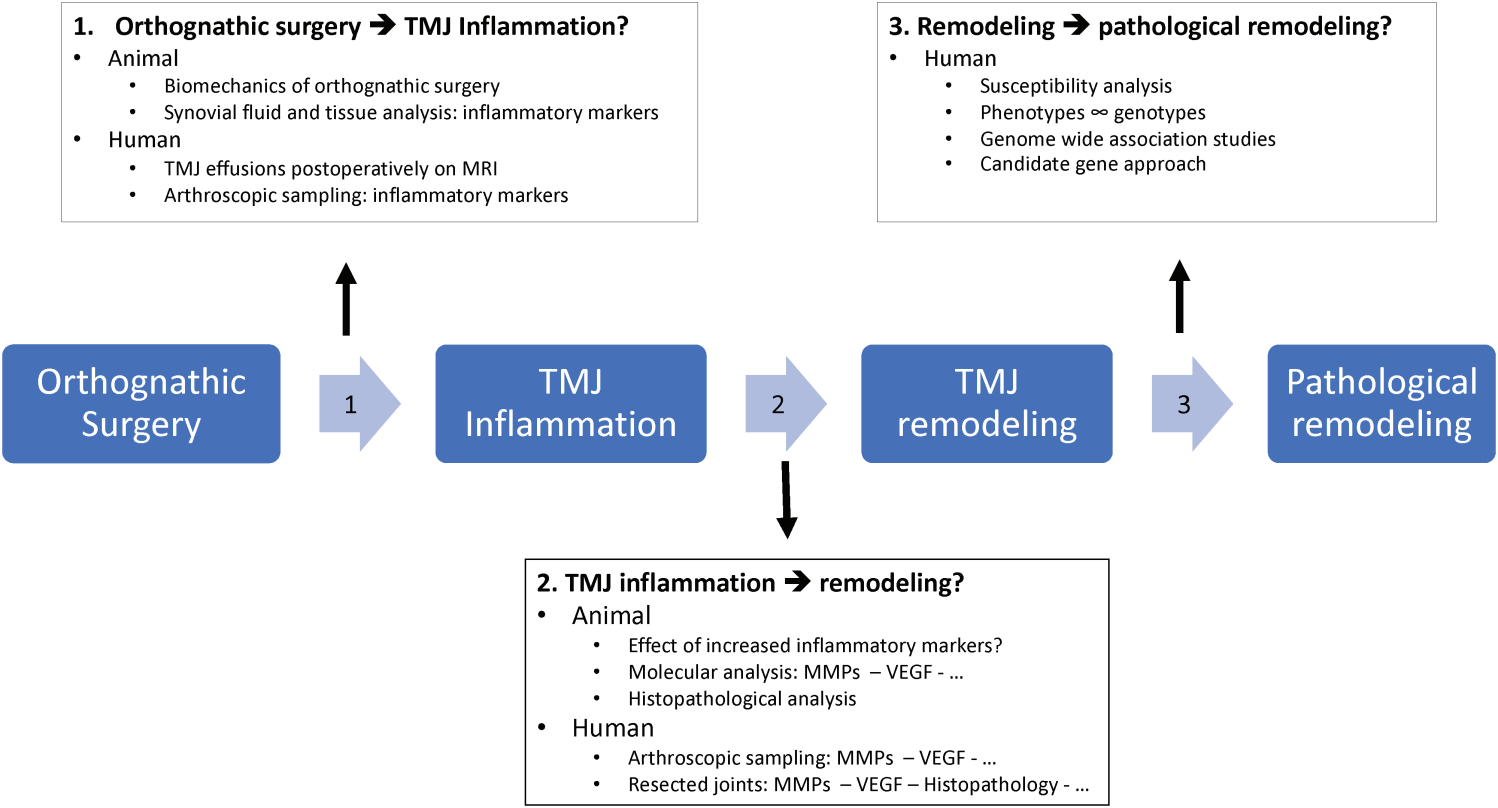
Infographic illustrating future research topics. A proposal of important future research directives and possible study designs: (1) how does orthognathic surgery cause TMJ inflammation, (2) how does inflammation cause TMJ remodeling, and (3) why do certain joints evolve in pathological remodeling.

Orthognathic surgery is an excellent approach for correcting dentofacial deformities. It produces good results in restoring occlusal and facial balance in patients with and without pre-existing TMJ problems. However, orthognathic surgery is believed to exert biomechanical stress at the TMJ, which leads to an inflammatory response and joint remodeling. Pathophysiological remodeling processes such as DJD or condylar resorption remain feared complications. This perspective study would like to bring up three fields of interest for future research on this topic (Figure 3):

1. Establishing a link between the biomechanical events of orthognathic surgery and the presence and amount of inflammation in the temporomandibular joint.
2. Identifying the pathways of how TMJ inflammation causes remodeling.
3. Identifying the cause and pathways of pathological remodeling.

The biomechanical stress caused by orthognathic surgery as illustrated in Figure 1 is believed to induce a biomechanical overload in the joint. The link between this biomechanical overload and the occurrence of inflammation is mostly opinion based. Future studies should investigate if and how orthognathic surgery causes inflammation in the joint. A first way of investigating this hypothesis is setting up observational studies in orthognathic patients. Inflammation in the TMJ can be established non-invasively by magnetic imaging resonance techniques with a focus on joint effusion, bone marrow edema and cartilage issues. These are however indirect signs of inflammation. Biochemical proof of inflammation can only be established by acquiring and analyzing synovial fluid or tissues. Synovial fluid can be screened for an upregulation of cytokines, which has been marked as an inflammatory parameter. Interleukin-1 (IL1), IL6, IL10, IL17, tumor necrosis factor-alpha, and the receptor activator of nuclear factor kappa-Β ligand are cytokines of interest. Enzyme-linked immunosorbent assays (ELISAs), multiplex bead array assays, and polymerase chain reaction (PCR) techniques have been advocated as analysis techniques in literature (Stenken and Poschenrieder, 2015). Synovial tissue analysis, a second approach, uses synovial tissue sampled by arthroscopic biopsy that is afterward analyzed for upregulation of cytokine-responsive genes. Where the first approach focuses on the mere presence of cytokines, the second one provides additional information of their effect. As not all orthognathic patients need invasive interventions in the TMJ, this raises ethical problems. Furthermore, one cannot experiment with omitting or adding different stressors as illustrated in Figure 1 in human patients to see which of them play a role in promoting TMJ inflammation. For the actual biochemical proof of inflammation following orthognathic surgery, animal studies seem more appropriate. The rat has been the model of choice for mechanical or chemical TMJ studies (Almarza et al., 2018). For extensive mechanical testing of joint structures, the larger rabbit is preferred. As performing orthognathic surgery on these animal models may be not feasible due to surgical access constraints, a larger sheep or (mini)pig model may be a suitable alternative. Furthermore, anatomically and functionally, the TMJ of a pig or minipig resembles the human TMJ the most. A convex shape of the condyle, biconcave articular disc, and combination of rotation and translation during motion are described in the pig TMJ. Other larger animal models, such as the sheep, have a slight differentiation in anatomy or function which makes extrapolating theories to the human TMJ a bit more difficult (Almarza et al., 2018). On the downside, using minipigs or pigs as an animal model has a higher operating cost which is why other models are frequently used.

If these types of studies have established the if’s and how’s of inflammation following orthognathic surgery, the next question needs to be answered. How does inflammation cause remodeling and why do certain joints transgress to pathological remodeling?

The link between inflammation and remodeling can be provided by extending the scope of the study design described in the previous paragraph. Once inflammation is induced by orthognathic surgery, one can wait until remodeling is observed on radiological examination. The animal condyles can then be further examined by histopathological and molecular analysis. ELISA and PCR techniques can be used to quantify cytokine (IL1, IL6, IL10, IL17, and TNF-α) as well as MMP (1, 2, 3, 9, and 13) levels. VEGF overexpression can be determined immunohistochemically. The same types of limitations as mentioned before apply when this kind of study needs to be performed on a human subject. However, literature shows that some patients with refractory DJD or condylar resorption are eventually treated with arthroscopy or more invasive procedures such as a TMJ alloprosthesis. Arthroscopic biopsy and synovial fluid collection can provide samples on which molecular analysis can be performed. Also, few to no studies report on histological or molecular analysis of the resected joints in DJD or condylar resorption patients. Histopathological and molecular analysis studies of these resected joints can be performed as has been done for example in condylar hyperplasia studies (Saridin et al., 2010; Vásquez et al., 2016, 2017). Cellularity, tissue layers, anatomical structures such as newly formed vessels or synovial hyperplasia can be characterized and so provide evidence of the aforementioned hypotheses.

As to determine why certain joints transgress to pathological remodeling, the answer probably can be found in the individual susceptibility analysis. Certain phenotypes are presumed to be more likely to develop degenerative joint disease or condylar resorption. Phenotype categories of interest are facial shape, skeletal configuration of the condyle and mandible, masticator forces, and biochemical actors of DJD and condylar resorption (interleukins, MMPs, VEGF, estrogen, BMPs). An interesting approach is to identify phenotypes that are present in patients who have developed pathological remodeling postoperatively and to link them to specific genotypes, as has been done for other TMJ pathology (Smith et al., 2019). Genome-wide association studies or candidate gene approaches can identify specific genetic variants that are associated with these phenotypes. This could lead to identification of actual cause of pathological remodeling but will also identify the molecular pathways that are involved.

If these molecular pathways have been confirmed, treatments interfering with these processes can be developed and tested using animal models, as listed above. Where DJD in animal models can be evoked using occlusion shifting techniques, the question remains how this can be done for condylar resorption. If any genetic cause of resorption could be identified, replicating this genetic variation in animals and combining mechanical overloading techniques could be an answer worth investigating. The final hurdle is to transfer this knowledge to human subjects, ultimately leading to clinically applicable treatment and prevention strategies.

## Author Contributions

P-JV, FV, and CP contributed to conception and design of the study. RJ and AL advised on specific radiological and clinical characteristics of the spectrum of remodeling. P-JV wrote the first draft of the study. P-JV, FV, RJ, AL, and CP wrote sections of the manuscript. All authors contributed to manuscript revision, and read and approved the submitted version.

### Conflict of Interest Statement

The authors declare that the research was conducted in the absence of any commercial or financial relationships that could be construed as a potential conflict of interest.
